# Association between Domains of the Clinical-Functional Vulnerability Index and Falls History in Older Adults: A Cross-Sectional Study

**DOI:** 10.3390/ijerph19137949

**Published:** 2022-06-29

**Authors:** Natália B. Moreira, Paulo C. B. Bento, Edgar Ramos Vieira, José L. P. da Silva, André L. F. Rodacki

**Affiliations:** 1 Departamento de Prevenção e Reabilitação em Fisioterapia, Rua Coronel H dos Santos, Jardim das Américas, 100-Centro Politécnico, Universidade Federal do Paraná, Curitiba 81530-000, Paraná, Brazil; nataliamoreira@ufpr.br; 2 Departamento de Educação Física, Rua Coronel H dos Santos, Jardim das Américas, 100-Centro Politécnico, Universidade Federal do Paraná, Curitiba 81530-000, Paraná, Brazil; p.bento063@gmail.com; 3 Department of Physical Therapy, Nicole Wertheim College of Nursing and Health Sciences, International University, Miami, FL 33199, USA; evieira@fiu.edu; 4 Departamento de Estatística, Rua Coronel H dos Santos, Jardim das Américas, 100-Centro Politécnico, Universidade Federal do Paraná, Curitiba 81530-000, Paraná, Brazil; jlpadilha@ufpr.br

**Keywords:** fall identification, risk factors, aged, frail elderly

## Abstract

Objectives: The study aimed to determine which domains, sets, and isolated or combined questions of the Clinical-Functional Vulnerability Index (CFVI-20) are associated with falls history in older adults. Methods: Instruments used were the CFVI-20 assessment and reported falls during the last year. The receiver operating characteristics (ROC) curves identified the performance of the CFVI-20 domains and questions in identifying older adults with and without falls history, while logistic regression identified relevant questions to identify fall history. Results: This study included 1725 individuals (71.9 ± 7.3 years). The area under the curve (AUC) between the CFVI-20 and fall history was 0.69. The mobility domain presented the largest AUC (0.71; *p* < 0.01), and most isolated domains showed low AUCs (0.51 to 0.58). Isolated questions were limited to identifying fallers. The regression analysis identified 7 questions of the CFVI-20 with falls. Conclusions: The CFVI-20 general score identified older adults with a fall history. When considered in isolation, most domains were limited to identifying falls, except for the mobility domain. Combining the CFVI-20 questions enabled identification of fallers.

## 1. Introduction

Accidents are the fifth-leading cause of death in older adults [1] and are the most typical injury mechanism [2]. Falls feature in more than half of fatal accidents and may result in fractures that cause immobility, disability, and hastened death [3]. The prevalence of falls indicates that 30 to 40% of community-dwelling older adults aged 60 or over experience at least one fall episode per year [4]. These figures increase to 32–42% for those over 70 years and up to 50% for those aged 80+ years [5,6]. In the United States, 3 million older adults are treated in emergency departments for fall injuries, representing significant health, social and economic problems [2].

Falls risk assessments needs to be multifactorial. Using a combination of instruments is required to comprehend the multidimensionality of falls to maximize the predictive ability [7]. Most falls risk assessment tools are not multidimensional. For example, a strong association between fall risk and frailty has been reported [1,8]. However, most falls risk assessments do not include a frailty evaluation, even though frail older adults are up to 3.6 times more prone to falls than their stronger counterparts [9]. Weakness, weight loss, slow gait, low physical activity levels, and exhaustion are traits of frailty and increased risk of falls [10].

In addition to frailty, other domains such as the cognition [11], health perception [12], fall awareness [4], polypharmacy [13], comorbidities [14], and psychosocial aspects [15] also play a relevant role in falls. Despite the vast number of instruments used to assess frailty (more than 50) [16], the instrument proposed by Fried and colleagues [17] is the most widely applied and is a substantial fall predictor [18]. However, Fried’s approach predominantly comprises physical aspects and does not incorporate the other relevant domains mentioned.

The Clinical–Functional Vulnerability Index–CFVI-20 [19] analyses eight domains (age, health self-awareness, functional status, cognition, humor, mobility, communication, and comorbidities) and has excellent clinimetric properties (i.e., internal consistency, equivalence, content validity, construct validity, sensitivity, and specificity) [16]. The CFVI-20 is a 40-point score that assesses most aspects approached by Fried (e.g., weight loss, walking speed, exhaustion) and includes other factors (e.g., cognition, comorbidities, polypharmacy, health perception) that may increase the ability to identify older adults with a fall history. For instance, cognitive impairments double the risk of falling [20]. Taking multiple medications (>3 drugs) is also a fall-related factor [13]. In addition, self-perceived health is a valid and reliable proxy of overall health status [21], which is associated with frailty [22].

The CFVI-20 presented a higher ability (11% increase/unit change) to identify older adults who had experienced a fall than did the Fried Phenotype [23]. However, it is unknown what domains of the CFVI-20 can identify older adults who experienced a fall. Identifying older adults with greater chances of falling is relevant as preventive measures can be proposed. Therefore, this study aimed to determine which domains, sets of domains and isolated or combined questions of the CFVI-20 are associated with falls history in older adults. It was hypothesized that it would be possible to identify which domains and combination of domains of the CFVI-20 would be related to older adults having a history of falls.

## 2. Methods

Participants aged 60 years or older living independently in the community were invited through Health Care Units disclosure from the County Health Secretary during their visit to the health services and by folders and flyers. A total of 1725 older adults agreed to participate in the study. They were aged between 60–96 years (71.0 ± 7.3 years), from which 1306 were women (76%; 70.8 ± 7.3 years), and 419 were men (24%; 71.5 ± 7.4 years). The number of participants represents 11.3% of the older adult population of Curitiba city, Brazil [24]. The sample size was estimated using the following parameters: (i) the population of 100,194 older adults; (ii) 95% confidence level; (iii) sampling error of 3%; (iv) 50% of anticipated falls prevalence, considering the maximum variance; (v) design effect of 1.5 to correct the sample selection biases; and (vi) 5% margin for losses and refusals [25]. Therefore, the initial estimated sample size necessary to answer our research question was 1664 older adults.

**Figure 1:**
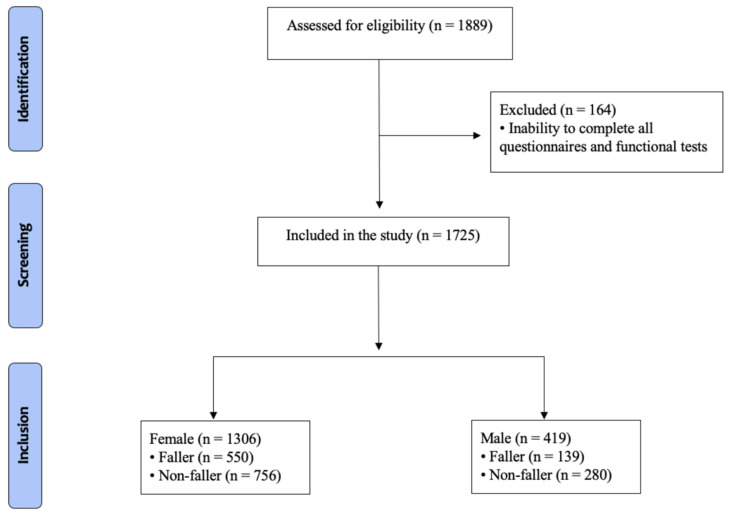
Flow chart.

The inclusion criteria were: (a) at least 60 years of age; (b) able to complete all tests, and (c) no cognitive impairments based on the Mini-Mental State Exam (MMSE) assessment that could impede the understanding of the CFVI-20 questionnaire after adjusting for the educational profile. The cutoff score for the MMSE was 20 points for illiterates, 25 for 1–4 years of education, 26.5 for 5–8 years of education, 28 for 9–11 years of education, and 29 for higher levels of education [26]. In addition, participants suffering from acute neurological and musculoskeletal problems, cardiovascular diseases, severe infections, and tumors were not included in the study. The University Ethics Committee (CAAE: 48548715.5.0000.5223) and the County Ethics Committee (CAAE: 48548715.5.3001.0101) approved the procedures, and all participants gave written consent before participating in the study. The participants were derived from a previous study [23]. The reporting of the study followed the Strobe guidelines (Figure 1).

A detailed description of the instruments and procedures can be found elsewhere [4]. Therefore, a short description is provided here. Participants completed a single 40 to 60 min evaluation session composed of questions related to sociodemographics (i.e., age, gender, body mass index, educational level, marital status, ethnicity, and economic class), fall episodes (i.e., a fall event in the past 12 months), clinical status (i.e., number of medicines), and frailty status assessment using the CFVI-20 instrument. A fall was defined as an unintentional event that resulted in changing the position to a lower level, irrespective of the resulting injury [27].

The CFVI-20 instrument has eight domains with 20 questions covering multidimensional aspects of older adults’ health: age (domain 1), health perception (domain 2), functional disabilities (domain 3), cognition (domain 4), mood (domain 5), mobility (domain 6), communication (domain 7), and comorbidities (domain 8) [19]. The higher the score (from 0 to 40 points), the higher the clinical-functional vulnerability and frailty. Scores between 0 and 6 points represent low vulnerability risk (not frail), from 7–14 moderate vulnerability risk (pre-frail), and equal to or greater than 15 high vulnerability risk (frail) [19].

### Data Analysis

Descriptive statistics (mean and standard deviation) were calculated to describe the participants’ demographics and physical characteristics. Initially, male and female participants were grouped to enable comparisons between falls history (fallers vs. non-fallers) regarding sociodemographics, clinical outcomes, and CFVI-20 scores using Kruskal–Wallis and Chi-square tests. Then, the area under the curve (AUC) of the receiving operating characteristics curve (ROC) was calculated considering the scores of each of the eight domains of the CFVI-20 to estimate the ability of each one to identify older adults with a fall history. The AUC of each question of the CVFI-20 was also calculated to determine which questions were relevant in identifying older adults with a fall history. Then, a logistic regression model was created considering the scores of all questions–except #16 (“Have you experienced two or more falls in the last year?”, not included due to its superimposed effect on the dependent variable related to falls). The logistic regression analysis performed had gender, marital status, ethnicity, economic class, and educational level as covariates. Finally, the regression equation model’s sensitivity and specificity were assessed using the AUC from the ROC curve. The ROC curve reveals the probability of an individual with a trait (e.g., fall history) to be correctly identified. The greater the discrimination performance, the closer the AUC is to 1. If the AUC is less than 0.5, the probability of identifying fallers from non-fallers is random. The significance level was set at *p* < 0.05, and all statistical procedures were performed using the SPSS statistical package (IBM, Armonk, NY, USA, version 22) and the R statistical software (R Foundation for Statistical Computing, Vienna, Austria, version 4.1.1) using the packages tidyverse, haven, and pROC.

## 3. Results

The prevalence of falls was 40%, from which female participants presented a greater prevalence than men (42 vs. 33%, respectively; *p* < 0.05). Overall, 848 participants were classified as low (50%), 586 as moderate (34%), and 291 (17%) as presenting high vulnerability risk. Marital status or ethnicity did not affect fall prevalence (*p* > 0.05). The history of falls was independent of age, cognition, and body mass index (*p* > 0.05). Fallers had lower levels of education than non-fallers. The fallers also presented lower economic status (40% vs. 43% female; 42% vs. 47% male) and higher prevalence of high vulnerability (31% vs. 9% females; 26% vs. 7% males) than non-fallers. The main characteristics of the participants are presented in Table 1.

The values between brackets represent the percentage within each group (fallers and non-fallers). The statistical comparisons refer to fall history and were performed after grouping participants by gender.

**Figure 2:**
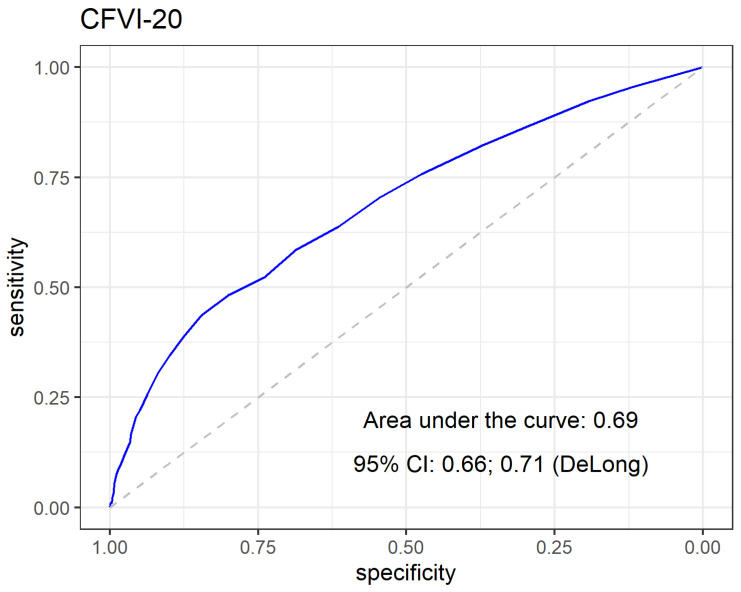
The receiving operating characteristics curve (ROC) and the area under the curve (AUC) to identify falls in older adults using the Clinical-Functional Vulnerability Instrument.

**Figure 3:**
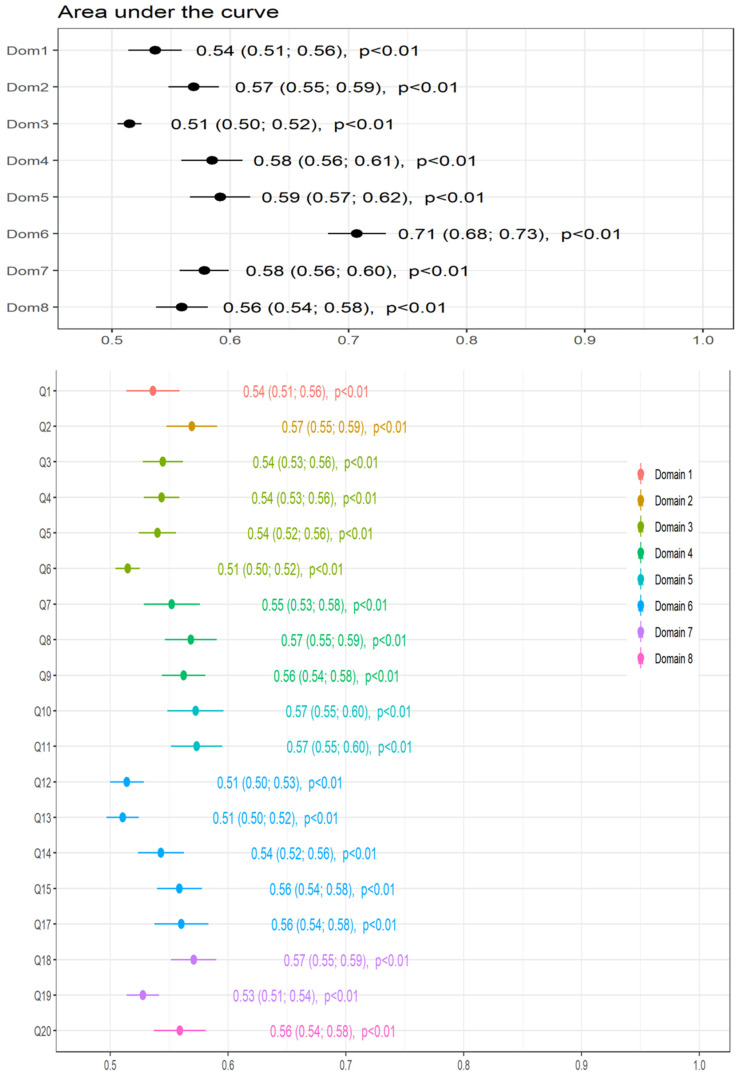
The area under the curve (AUC) from the receiving operating characteristics curve (ROC) for the scores of each domain (upper panel) and each question (lower panel) of the CFVI-20 questionnaire. Question 16 was not included due to its superimposed effect on the dependent variable.

The AUC of the ROC for the CFVI-20 total score vs. falls history was 0.69 (CI 0.66; 0.70, *p* < 0.01; Figure 2). The AUCs of the eight domains of the CFVI-20 ranged from 0.51 (i.e., daily living activity; domain 3) to 0.71 (i.e., functional mobility; domain 6). Interestingly, the AUC for domain 6 (mobility) regarding falls ranged from 0.51–0.56 (questions 12 to 17). The AUC of question 12 was close to the identity line (AUC = 0.50; “Can you raise your arms above shoulder level?”), while the AUC of question 13 (“Are you able to handle and hold small objects?”) was also unable to identify faller. Figure 3 shows the AUC of the scores of the eight domains (upper panel) and each question (lower panel) of the CFVI-20 questionnaire.

Finally, all questions were used as explanatory variables in the logistic model to observe the questions with the most significant possibilities to identify fallers and non-fallers. The model also considered explanatory covariates (i.e., sex, marital status, ethnicity, economic class, and educational level). The regression model output and the contribution of the questions and covariables are indicated in Table 2.

The older adults with vision problems presented 1.84 times more chance of experiencing a fall; health perception and mental capacity to control the finances and remembrance also played a relevant role (1.34, 1.59, and 1.54 times, respectively). Those suffering from walking difficulties showed 1.46 times more chance of experiencing a fall than those with no problems. With polypharmacy, comorbidities, and recent hospitalizations, older adults showed comparable odds ratios to falls (1.30 times). Finally, male participants presented lower odds of having experienced a fall than their female counterparts. The other covariates were not statistically significant to the model.

## 4. Discussion

Falls prevalence was comparable to that described by the World Health Organization [28], which reported 32–42% for those above 70 years. The current literature confirmed that females are more susceptible to falls than males [29]. Older women have greater levels of disability and are more susceptible to falls and fall-related injuries than older men [30]. Exhaustion and weakness are more prominent in women than in men. Women have a greater prevalence of frailty and vulnerability than men [31].

The prevalence of high vulnerability (17%) was slightly lower than previously found (20%) [32]. The prevalence of moderate vulnerability (34%) was similar to that previously found among Brazilian older adults, with scores ranging from 31% to 39% [33]. Additionally, older adults with a fall history showed a higher vulnerability prevalence than those with no falls, irrespective of sex (females 31% vs. 9%; males 26% vs. 7%).

The ability of the CFVI-20 to identify individuals with previous falls was comparable to that previously reported (~0.69) [23]. The Study of Osteoporotic Fractures and the Fried frailty indexes had slightly smaller AUCs (0.63) in identifying recurrent fallers [34]. The more comprehensive nature of the CFVI-20 questionnaire (i.e., a broader set of domains) may have captured other fall-related aspects and surpassed the predominant focus on physical components and functional elements that are primarily emphasized in other frailty instruments resulting in higher accuracy in identifying fallers.

When the domains of the CFVI-20 were analyzed separately, it was observed that most failed to discriminate fallers from non-fallers. The largest AUC was obtained from the mobility domain. However, there is a superimposed effect, as fall recurrence (i.e., more than one fall) is included in the mobility domain. The significant number of older adults with recurrent falls (~43% fell more than once) may explain why this domain was the most superior at identifying fallers among the other domains of the CFVI-20.

Interestingly, no isolated question was able to identify fallers. This reinforces the concept that fall are multidimensional and fallers can not be identified from isolated tests or domains. On the other hand, the mobility domain was expected to better identify fallers, as it has been considered one of the most relevant fall-related aspects [35,36]. Although the CFVI-20 domain refers to mobility (questions 12 to 17), only a few aspects are devoted to explaining one’s capacity to move independently. For instance, question 12 asks if one can raise their arms above their shoulder level, while question 13 refers to handling and holding small objects. Both questions are closely associated with the upper segments and are relatively limited in expressing mobility. Indeed, the performance of upper limb muscles is unlikely to explain mobility, which is more related to lower limb locomotor muscles. Furthermore, upper limb performance presents a weak-to-negligible correlation with the functional mobility [37]. In addition, mobility may not be directly related to incontinency (question 17), which was also included in the mobility domain in the CVFI-20 instrument. The only question directly related to mobility (question 15) is the time to walk 4 m, which may not entirely reveal the most relevant mobility aspects when discriminating fallers from non-fallers. Although walking speed is deemed as an excellent predictor of falls for older adults, a non-linear relationship has been described between falls and the speed in the 4-m walk test in which the fastest (>1.3 m·s^−1^) and the slowest (<0.6 m·s^−1^) had more falls than normal gait speeds [38]. Perhaps other tests (e.g., timed up and go test—TUG) may be more specific to determining mobility and more suitable for identifying and predicting falls [39].

The regression equation that was performed using separated questions of the CFVI-20 questionnaire identified that older adults with vision problems are 1.8 times more prone to have experienced a fall. These findings align with reports that vision issues double the risk of falling [40]. In addition, vision problems may impair balance and increase the risk of falls [6]. Older adults with vision difficulties may have problems with contrast, depth perception, and a reduced ability to negotiate obstacles [41].

Health self-perception (question 2) and the consequences of dealing with finance (question 4) were also relevant fall-related aspects. Fall history prevalence was 1.34 times greater in older adults that reported regular or bad health self-perception and 1.59 times greater in those who reported difficulties in managing financial aspects due to health-related issues. It has been demonstrated that negative self-perceptions of health, age, and aging predict worsening health and mortality [42]. In addition, negative self-perception of health is also associated with fear of falling, which has been reported as being related to older adults’ fall history [43].

Cognition declines and several self-reported depressive symptoms (sadness, hopelessness, lack of motivation) have been associated with falls among older adults [44]. Hofmann and colleagues indicated that depressive symptoms were related to short-term fall risks, with a 0.5 SD increase representing a 30% increase in fall risk [45]. It indicated that variability in depressive symptoms also played a role. Our results are in the same direction and suggest that older adults suffering from depressive symptoms (question 10) are 35% more likely to have experienced a fall than those without it.

Additionally, multimorbidity [14], polypharmacy [13], and recent hospitalizations were among the questions related to fall history. Health status perception and clinical conditions are relevant in mediating falls in older adults. Older adults with a falls history and fear of falling may have negative health self-perception and lower self-confidence regarding functional independence. They might be incapable of taking care of themselves, must rely on others, and become a burden to family or society.

Finally, walking difficulties to perform activities of daily living can impact fall risk (question 15), and older adults unable to walk 4 m in under 5 s (<0.8 m·s^−1^) were 1.46 times more likely to have experienced a fall. It has been demonstrated that walking characteristics are of great importance when analyzing physical performance and the risk of falling [46], and gait speed has been considered a standard clinical evaluation described as the “sixth vital sign” [6,36] and a health and functional marker of aging and disease [6,47].

The present findings suggested that the CVFI-20 questionnaire general score is a feasible instrument for detecting and better identifying fallers than other frailty instruments. The ROC curves indicated that the instrument’s accuracy (AUC = 0.69) was borderline and was assumed as moderate. On the other hand, isolated domains presented limited ability to detect falls, except for the mobility domain due to its superimposed effect concerning the outcome variable. Studies that combined or integrated a number of domains also failed to identify fall occurrences in older adults. For instance, Cardon-Verbecq and colleagues combined physical and mental domains using the cognitive timed up and go test and could not identify the fallers [10]. However, a broader set of domains were not applied. Combining domains (e.g., physical, health, cognition, social, perceptive, and psychological) is a promising strategy for providing more comprehensive fall identification and prediction assessments. Some studies have indicated that several domains are closely related to falls, although a general hierarchical order has not yet been established. The arguments proposed by Ensrud and colleagues that frailty may be a marker for other conditions that influence the risk of falls may explain why single or even combining a small number of factors or domains failed to explain fall occurrences [9]. The significant emphasis placed on physical domains seems a limiting aspect when identifying fallers among older adults. It has been advocated that some physical tests are relatively limited at predicting falls and are recommended to be applied in conjunction with other tests to reveal the dependence on multiple intrinsic and extrinsic factors [48]. Thus, instruments incorporating a comprehensive range of aspects derived from the most relevant domains may increase fall identification in older adults. Identifying the most relevant domains and the respective tests remains challenging due to the multifactorial nature of a fall.

### Strengths and Limitations

The strengths of our study include the use of clinical and accessible instruments to evaluate the frailty and fall occurrence in a large sample size of community-dwelling older adults, which amplifies the clinical application of the study findings. The reader should also consider some limitations. First, we did not analyze recurrent falls. Second, the cross-sectional design precluded investigating the time-dependent relation between variables. For instance, it is not possible to distinguish if a fall caused functional declines or if it was related to frailty. Longitudinal studies are required to identify such aspects. Third, the majority of the sample comprised older adults with low vulnerability prevalence. Fourth, the evaluation did not specify the use of fall-risk-increasing drugs (e.g., sedatives and hypnotics). The exact number of falls caused by drugs is unknown because falls are not officially recognized as adverse reactions. Other studies are required, including among older adults with significant limitations, different settings, and clinical aspects. It is recognized that differences between genders may also be considered in future studies. Finally, predictive studies are also needed as a preventive tool.

## 5. Conclusions

The general score of the CFVI-20 instrument presented a greater capacity to identify fallers than other frailty instruments. On the other hand, most domains of the CFVI-20 showed a limited ability to identify falls, except the mobility domain. The superimposed effect of falls on the mobility domain may have identified fallers better than in other domains. When the CFVI-20 questions were assessed individually to identify fallers, it was observed that most showed little possibility of distinguishing fallers from non-fallers, as evidenced by the low AUCs. The regression analysis indicated that combining questions significantly identified older adults with a fall history. Identifying fallers and non-fallers is a complex task that requires one to consider multiple domains. The finding that mobility is more relevant to identifying fallers among older adults is trivial; however, it may be complemented by other domains. Therefore, additional studies combining physical, health, cognition, social, perceptive, and psychological aspects may identify older adults with and without a fall history.

## Figures and Tables

**Table 1:** Sociodemographic, clinical characteristics, and CFVI-20 data stratified by gender and falls history (*n* = 1725).

		Females (*n* = 1306)		Males (*n* = 419)		*p*
		Faller (*n* = 550)	Non-Faller (*n* = 756)	Faller (*n* = 139)	Non-Faller (*n* = 280)	
**CFVI-20 Sociodemographic and clinical characteristics**	Age (years)	70.6 ± 7.4	71.0 ± 7.3	71.3 ± 7.2	71.4 ± 7.2	0.254
	Body Mass Index (kg/m^2^)	28.0 ± 5.3	28.2 ± 5.0	28.3 ± 5.2	28.2 ± 4.9	0.704
	Educational level	*n* (%)	*n* (%)	*n* (%)	*n* (%)	
	Illiterate	58 (10)	56 (7)	14 (10)	22 (8)	0.001
	Primary complete	259 (47)	293 (39)	57 (41)	93 (33)	
	Secondary complete	163 (30)	286 (38)	49 (35)	118 (42)	
	College complete	70 (13)	121 (16)	19 (14)	47 (17)	
	Marital Status					
	Married	196 (36)	275 (36)	84 (60)	185 (66)	0.903
	Widowed	248 (45)	298 (39)	22 (16)	28 (10)	
	Divorced	57 (10)	105 (14)	22 (16)	42 (15)	
	Single	49 (9)	78 (10)	11 (8)	25 (9)	
	Ethnicity					
	Caucasian	429 (78)	581 (77)	103 (74)	220 (79)	0.732
	Mulatto/African	103 (19)	141 (19)	32 (23)	51 (18)	
	Asiatic	18 (3)	34 (4)	4 (3)	9 (3)	
	Economic class					
	High (A + B)	218 (40)	322 (43)	58 (42)	133 (47)	<0.001
	Middle (C)	260 (47)	366 (48)	54 (39)	99 (35)	
	Low (D + E)	72 (13)	68 (9)	27 (19)	48 (17)	
	Low	181 (33)	430 (57)	55 (40)	182 (65)	<0.001
	Moderate	200 (36)	260 (34)	48 (34)	78 (28)	
	High	169 (31)	66 (9)	36 (26)	20 (7)	

**Table 2:** Questions included in the adjusted regression equation model selected to identify older adults with and without fall history.

Explanatory Variables	Beta	OR	IC	*p*
Question 2 (Regular or bad) (How do you feel about your health?)	0.31	1.34	1.06; 1.73	0.014
Question 4 (Yes) (Do you stop controlling your finances because of your health?)	0.46	1.59	1.10; 2.29	0.013
Question 9 (Yes) (Do you forget things that impede you from performing daily activities?)	0.43	1.54	1.16; 2.05	0.003
Question 10 (Yes) (In the last month, did you feel unmotivated, sad, and hopeless?)	0.30	1.35	1.09; 1.66	0.005
Question 15 (Yes) (Do you have walking difficulties that impede performing daily routines?)	0.38	1.46	1.10; 1.94	0.008
Question 18 (Yes) (Do you have vision problems that impede performing daily activities?)	0.61	1.84	1.40; 2.41	<0.001
Question 20 (Yes) (Do you have five or more illnesses; use five or more medicines daily; recent hospitalization)	0.26	1.30	1.03; 1.64	0.024
Gender (Male)	−0.31	0.735	0.58; 0.94	0.013

Beta—regression coefficient; OR—odds ratio; IC—interval of confidence.

## Data Availability

The data can be obtained from authors upon request.
